# Extending the Transdiagnostic Model of Attachment and Psychopathology

**DOI:** 10.3389/fpsyg.2016.00484

**Published:** 2016-03-31

**Authors:** Tsachi Ein-Dor, Dina Viglin, Guy Doron

**Affiliations:** School of Psychology, Interdisciplinary Center HerzliyaHerzliya, Israel

**Keywords:** transdiagnostic model, attachment, psychopathology, insecurity, internalizing, externalizing, thought disorder spectrum

## Abstract

Research has suggested that high levels of attachment insecurities that are formed through interactions with significant others are associated with a general vulnerability to mental disorders. In the present paper, we extend Ein-Dor and Doron’s (2015) transdiagnostic model linking attachment orientations with internalizing and externalizing symptoms, to include thought disorder spectrum symptoms. Specifically, we speculate on the processes that mediate the linkage between attachment insecurities and psychosis and obsessive compulsive disorder (OCD) symptoms, and indicate the different contexts that might set a trajectory of one individual to one set of symptoms while another individual to a different set of symptoms.

According to the World Health Organization (WHO), “untreated mental, neurological, and substance use disorders exact a high toll, accounting for 13% of the total global burden of disease.” In recent reports, the WHO has highlighted the need to find new ways to understand psychopathology and combat its repercussions (World Health Organization [WHO], 2012). It was previously argued that the ontogeny and maintenance of psychopathology is heavily influenced by one’s history of interactions with other people, specifically in times of need (Bowlby, 1980; Sroufe et al., 2004; Sroufe, 2005) and on the attachment dispositions that develop during these times. Consistent with this, Ein-Dor and Doron’s (2015) have proposed a transdiagnostic model on the effects of attachment dispositions on psychopathology including internalizing (i.e., mood and anxiety disorders, such as major depression, generalized anxiety disorder, panic disorder, and social phobia; Krueger and Markon, 2006, 2011) and externalizing symptoms (i.e., substance and antisocial disorders; Krueger and Markon, 2006, 2011). In the current paper, we expend Ein-Dor and Doron’s (2015) model to include the third primary dimensions of psychopathology – thought disorder spectrum (i.e., psychotic symptoms and obsessive–compulsive disorder; Caspi et al., 2013; Kotov et al., 2011a,b).

## Attachment Theory and Psychopathology

According to Bowlby (1982), when people, particularly close others, habitually respond sensitively to our needs and grant us support and care, we develop a trait-like sense of attachment security (see Mikulincer and Shaver, 2007 for an extensive review). The residues of these experiences with others during times of need are stored as mental representations of self, others, and the world, which Bowlby (1973) called *internal working models*. These representations shape expectations about others’ availability and responsiveness, and organize strategies for coping with threats and for regulating negative emotions. When other people are habitually unavailable, unreliable, or rejecting of bids for support and care, a person may become chronically insecure with respect to close relationships and adopt insecure attachment dispositions.

Attachment theory, therefore, relates to the activation of an innate psychobiological system (*the attachment behavioral system*) that motivates people to seek proximity to significant others (*attachment figures*) when they need protection from threats. Social and personality psychologists generally conceptualize adult attachment patterns as regions in a continuous two-dimensional space (e.g., Brennan et al., 1998). The dimension of attachment-related anxiety reflects the extent to which a person worries that others will not be available or helpful in times of need. Anxious individuals exaggerate their sense of vulnerability and insistently call on others for help and care, sometimes to the point of being intrusive (e.g., Feeney and Noller, 1990). The second dimension, attachment-related avoidance, reflects the extent to which a person distrusts relationship partners’ goodwill, strives to maintain independence, and relies on deactivating strategies for dealing with threats and negative emotions (e.g., Fraley and Shaver, 1997). Attachment security is defined by low scores on both anxiety and avoidance. Secure people generally cope with threats by relying on internal resources developed with the help of security-enhancing attachment figures or by effectively seeking support from others or collaborating with them (Shaver and Mikulincer, 2002).

Indeed, research has indicated that high levels of attachment insecurities are associated with a general vulnerability to mental disorders including anxiety disorders (generalized anxiety disorder, social phobia, panic with/without agoraphobia; e.g., Schimmenti and Bifulco, 2015), obsessive-compulsive disorder (e.g., Doron et al., 2012), post-traumatic stress disorder (e.g., Ein-Dor et al., 2010), eating disorders (e.g., Illing et al., 2010), and depression (e.g., Catanzaro and Wei, 2010). Attachment insecurity has also been linked with many personality disorders (Meyer and Pilkonis, 2005; Crawford et al., 2007).

Ein-Dor and Doron’s (2015) have argued, however, that attachment theory has difficulty simultaneously explaining the mechanisms by which attachment insecurities lead to multiple disorders (i.e., the question of multifinality; Cicchetti, 1984; Egeland et al., 1996), and why one individual with a particular attachment orientation develops one set of symptoms while another with the same attachment vulnerability develops another set of symptoms (i.e., the question of divergent trajectories; Nolen-Hoeksema and Watkins, 2011). To bridge this gap, they proposed a transdiagnostic model of attachment insecurities.

## Ein-Dor and Doron’s (2015) Transdiagnostic Model of Psychopathology

Ein-Dor and Doron’s (2015) model refers to (a) the mechanisms by which attachment dispositions (i.e., the transdiagnostic factors) may cause the different disorders they are associated with (i.e., the mediated pathways underpinning multifinality), and (b) why a given disposition may lead to different disorders in different people or to different disorders within the same person over time (i.e., divergent trajectories).

## Mechanisms Linking Attachment Dispositions to Multiple Psychopathological Disorders (i.e., Multifinality)

Research and theory have indicated that people high on attachment anxiety tend to adopt hyperactivating attachment and emotion-regulation strategies (i.e., energetic, insistent attempts to obtain care, support, and love from others) as a means of regulating distress and coping with threats (Mikulincer and Shaver, 2003). They are also inclined to exaggerate appraisals of threats (e.g., Mikulincer et al., 2000), to have difficulties in suppressing negative thoughts and feelings (e.g., Mikulincer et al., 2004), and to ruminate on distressing thoughts (Mikulincer and Florian, 1998).

According to Ein-Dor and Doron’s (2015) transdiagnostic model, attachment anxiety may increase vulnerability to psychopathology by a Dark Triad of processes: (a) maladaptive emotion regulation processes, with a tendency to upregulate negative affectivity; (b) greater vigilance to threat-related cues and heightened empathic accuracy; and, (c) a lower level of perceived others responsiveness – seeing others as less responsive and supportive and less understanding to one’s needs (a broader notion than Reis and colleagues’ perceived partner responsiveness; e.g., Einstein, 1931). This Dark Triad of people high in attachment anxiety – intensified negative affectivity, hypervigilance to threats, and low perceived others responsiveness – might constitute the proximal transdiagnostic risk factors (see Nolen-Hoeksema and Watkins, 2011; also called intermediate phenotypes) that mediate the relationships between attachment anxiety and multiple psychopathologies, and that launch anxious individuals on pathways that are probabilistically related to various psychopathological outcomes.

Regarding attachment avoidance, theory and research have indicated that it is organized around deactivating strategies of affect regulation, which involve de-emphasizing threats and trying to cope with them alone, without seeking help or support from other people (e.g., Kobak et al., 1993; Shaver and Mikulincer, 2002). Avoidant people also deny attachment needs and suppress attachment-related thoughts and emotions (Mikulincer and Shaver, 2003). According to the Ein-Dor and Doron’s (2015) transdiagnostic model, these tendencies may be the initiating conditions for a second Dark Triad of processes that link attachment avoidance with multiple psychopathological disorders: (a) maladjusted emotion regulation processes, with a tendency to downregulate affectivity and employing distancing strategies; (b) compulsive self-reliance; and (c) lower levels of social support and perceived others responsiveness. As with attachment anxiety, the dark triad of people high in attachment avoidance – cognitive and emotional avoidance, compulsive self-reliance, and low perceived others responsiveness – might comprise the proximal transdiagnostic risk factors that mediate the relationships between attachment avoidance and multiple psychopathologies.

## Moderators of the Effects of Proximal Risk Factors

**FIGURE 1:**
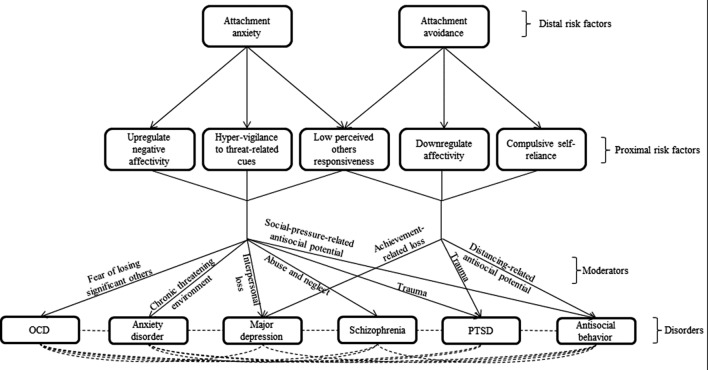
**In this transdiagnostic model, attachment dispositions serve as distal risk factors for multiple psychopathological disorders.** Each disposition affects a triad of proximal risk factors, which mediate the effect of attachment anxiety and avoidance on psychopathology. Specific moderators determine the divergent trajectories that individuals high on the proximal risk factors may take.

The moderators in Ein-Dor and Doron’s (2015) transdiagnostic model determine what particular symptoms proximal transdiagnostic risk factors will lead to in a given individual. Moderators create symptoms by (a) raising concerns or themes that proximal risk factors then act upon, (b) shaping responses through conditioning, or (c) determining the reinforcement value of certain stimuli (Nolen-Hoeksema and Watkins, 2011). For example, one possible moderator is chronic *mild-to-moderate* threatening environment (e.g., living in a rough neighborhood, facing a constant but mild political violence, or living under prolonged family related conflicts). In such an environment, emotions of fear and anxiety often arise (LeDoux, 2000). The tendency of people high on attachment anxiety to be emotionally overreactive and hyperattentive to threats would exacerbate and maintain these feelings of anxiety and fear. At the same time, their low perceived others responsiveness would hinder an effective alleviation of these feelings by the aid of supportive others. As a result, the likelihood of developing an anxiety disorder may increase. In contrast, the cognitive and emotional distancing strategies of people high on attachment avoidance may shield them from mild-to-moderate feelings of anxiety and fear, and, therefore, reduce the likelihood of developing anxiety-based disorders under such chronic *mild-to-moderate* threatening environment. We believe that Ein-Dor and Doron’s (2015) model may also be extended to include thought-disorder-spectrum-related symptoms. The extended model is presented in **Figure 1**.

## Attachment and Thought Disorder Spectrum

Thought disorder spectrum refers to a general liability toward symptoms of psychosis and obsessive-compulsive disorder (Kotov et al., 2011a,b; Caspi et al., 2013). It constitutes syndromes characterized by disordered thoughts such as schizophrenia spectrum disorders (schizophrenia, other psychotic disorders, and schizotypal personality disorder) and obsessive compulsive disorder (OCD). In the current review, we focus on psychotic disorders and OCD.

### Psychotic Disorders

Bowlby (1980) contended that distancing defenses, which are usually employed by people high on attachment avoidance, might involve the exclusion of specific events and representations of others from consciousness and create “segregated” mental systems that preclude a stable and coherent sense of identity. During interactions with a frightened, threatening, and/or emotionally disconnected parent, insecure children experience their parents in secluded ways: sometimes as caring and available, at other times as frightened, and at still other times as frightening (Liotti, 1992; Hesse and Main, 2006). As a result, they develop multiple incompatible working models of self that cannot be integrate into a coherent, meaningful inner life. Liotti (1992) has contended that this kind of difficult attachment experience and the lack of inner coherence and integration heighten the risk for dissociative experiences underlying psychosis related positive symptoms (e.g., hallucinations; Moskowitz et al., 2009).

Studies on patients diagnosed with psychosis have indicated that a diagnosis of psychotic disorders was associated with insecure states of mind in the adult attachment interview (AAI; Hesse, 1999) as compared with patients diagnosed with affective disorders (Dozier et al., 1991). Insecure attachment was also related to paranoid thoughts among adolescents with early psychosis as compared with controls (Korver-Nieberg et al., 2013) and with worsened indicators of recovery as compared with participants with HIV/AIDS who had no history of experiencing severe mental illness (Ringer et al., 2013).

Studies in non-clinical samples have indicated that higher ratings of attachment insecurity (both anxiety and avoidance) on self-report scales are associated with more severe psychotic symptoms (Mickelson et al., 1997), with higher scores on “schizophrenism” (reflecting the prevalence of bizarre beliefs) and “anhedonia” (social withdrawal and loss of pleasure; Wilson and Costanzo, 1996), and with positive (mainly anxiety) and negative (mainly avoidance) schizotypy symptoms (Sheinbaum et al., 2013). Attachment anxiety and avoidance also mediated the effects of neglect on paranoid beliefs, whereas attachment anxiety but not avoidance, the effects of sexual abuse on hallucinations (Sitko et al., 2014).

### Obsessive Compulsive Disorder (OCD)

Bowlby (1973, 1982) has contended that the attachment behavioral system emerged as an adaptation over the course of mammalian evolution. Because human infants are born immature and require a long period of care and protection, they are equipped with a repertoire of behaviors and action tendencies that increase the likelihood that they will remain proximal to supportive others, and, thereby, safe and secure. People high on attachment anxiety tend to hyper activate the attachment system, to continuously monitor their surrounding for threats, and to be constantly afraid of being separated or abandoned by people close to them, either because some harm will befall them or because they themselves might inflict harm upon their close ones. These tendencies are based on the adaptive advantage of being able to “foresee” the behavior of other people, to anticipate future scenarios (both social and non-social) and to adjust one’s behavior in order to make the environment more predictable and secure (Abed and de Pauw, 1998). OCD, which characterized by a dire need to acquire control over uncontrollable events, may be seen as an extreme case of these adaptive tendencies (Gilbert, 2001; Brune, 2006; Moulding and Kyrios, 2006). Overly anticipating future threats or a biased anticipation toward the negative consequences of one’s own thoughts and intentions in a search for control and safety may lead to a constraint of behavioral flexibility which may become an OCD (Saxena and Rauch, 2000; Doron et al., 2015).

Studies on patients diagnosed with OCD have indicated that they were higher on attachment anxiety, but not on avoidance, than patients diagnosed with other anxiety disorders (Doron et al., 2012), major depression (Shaker and Homeyli, 2011) or community controls (Doron et al., 2012). Studies in non-clinical samples have corroborated these clinical findings and indicated that higher ratings of attachment anxiety on self-report scales are associated with more severe OCD symptoms (Doron et al., 2009, 2012a,b; Koohsar and Bona, 2011), and that attachment anxiety mediated the links between maladaptive parental care and obsessive beliefs (e.g., responsibility, threat estimation, perfectionism, and uncertainty; Yarbro et al., 2013).

The developmental trajectory of OCD with respect to early attachment dispositions has yet to be directly examined. With that being said, longitudinal research has indicated that attachment anxiety in the Strange Situation at 12 months of age was linked with greater prevalence of various anxiety disorders (among which was OCD) at age 17 (Warren et al., 1997).

## Moderators of the Effects of Proximal Risk Factors on Thought Disorder Spectrum

One major moderator that may set the path to the development of OCD and thought related disorders is fear of losing significant others or harming them (as opposed to actual loss). This fear may bring about overinflated sense of responsibility, intolerance for uncertainty and related moral and relationship concerns (Doron et al., 2007, 2008, 2013). These, in turn, fuel rumination and intrusive thoughts as well as the unending efforts and repetitive behaviors aimed at reducing the associated anxiety. Because loss is eventually unavoidable, the person may be caught in a vicious cycle of harm avoidance, which may consolidate into OCD.

The experience of repetitive abuse and emotional neglect from close others was reliably linked with disorganized attachment (see van Ijzendoorn et al., 1999 for a meta-analysis) early in life, but may also function as a moderator later in life. Indeed, abuse and emotional neglect were linked with liability for dissociative symptoms and psychosis (see Morgan and Fisher, 2007 for a critical review). Thus, child abuse and neglect may cause the severance of self into unintegrated representations, the breakdown of the attachment behavioral system, and, as a result, the crackup in thinking processes and disturbances in emotional responses. Such disassociation between attachment representations, with all their detrimental consequences on thought processes and affect regulation, may also negatively affect the quality of interpersonal relationships that often appears as the first symptom in the onset of psychosis (Schimmenti and Caretti, 2016). A history of repetitive abuse and emotional neglect may create a predisposition for psychosis (Read et al., 2008), and when combined with an acute stresor in adulthood, of various types, may elicit dissociative symptoms and psychosis (thereby, repetitive abuse and emotional neglect may serve as moderators for psychopathology and as distal risk factors). In addition, abuse and neglect may bring about co-morbidity of depression (co-morbid with psychosis and OCD) because they hinder the formation of basic and later interpersonal trust (and foster hopelessness), and/or anxiety disorders [specifically, Generalized Anxiety Disorder (GAD) and PTSD] because of the inability to disengage from the threatening stimuli.

## Concluding Comments

Ein-Dor and Doron’s (2015) proposed a transdiagnostic model of psychopathology addressing two open questions with regards to the links between attachment and psychopathology. What are the mechanisms by which attachment dispositions cause all the different disorders they are associated with, and why a given disposition leads to different disorders in different people or to different disorders within the same person over time. They proposed that each attachment orientation has its own dark triad of processes that link it to psychopathology, and that by interacting with a specific moderator they launch an individual on a pathway for a particular disorder. In this paper, we extended this model by applying it to the thought disorder spectrum. We hope that this extension of the model will allow for a more comprehensive and efficient view on the developmental trajectory linking early environmental influences with adult psychopathology.

## Author Contributions

GD together with the first author, TE-D, wrote the first draft of the paper. DV edited the first draft.

## Conflict of Interest Statement

The authors declare that the research was conducted in the absence of any commercial or financial relationships that could be construed as a potential conflict of interest.
